# Cyclic ACTH-secreting thymic carcinoid: a case report and review of the literature

**DOI:** 10.20945/2359-3997000000346

**Published:** 2021-04-12

**Authors:** Elisa B. Lamback, Sérgio Altino de Almeida, Ricardo Terra, Carlos Gil Ferreira, Vera Luiza Capelozzi, Rui Haddad, Mônica R. Gadelha

**Affiliations:** 1 Universidade Federal do Rio de Janeiro Faculdade de Medicina e Hospital Universitário Clementino Fraga Filho Unidade de Medicina Interna e Serviço de Endocrinologia Rio de Janeiro RJ Brasil Unidade de Medicina Interna e Serviço de Endocrinologia, Faculdade de Medicina e Hospital Universitário Clementino Fraga Filho, Universidade Federal do Rio de Janeiro, Rio de Janeiro, RJ, Brasil.; 2 Instituto Estadual do Cérebro Paulo Niemeyer Unidade de Neuroendocrinologia Rio de Janeiro RJ Brasil Unidade de Neuroendocrinologia, Instituto Estadual do Cérebro Paulo Niemeyer, Rio de Janeiro, RJ, Brasil.; 3 Instituto Estadual do Cérebro Paulo Niemeyer Laboratório de Neuropatologia e Genética Molecular Rio de Janeiro RJ Brasil Laboratório de Neuropatologia e Genética Molecular, Instituto Estadual do Cérebro Paulo Niemeyer, Rio de Janeiro, RJ, Brasil.; 4 Centro de Imagem Copa D'Or e Hospital Copa Star Divisão de Medicina Nuclear Rio de Janeiro RJ Brasil Divisão de Medicina Nuclear, Centro de Imagem Copa D'Or e Hospital Copa Star, Rio de Janeiro, RJ, Brasil.; 5 Rede D'Or São Paulo e Hospital Copa Star Unidade de Cirurgia Torácica Rio de Janeiro RJ Brasil Unidade de Cirurgia Torácica, Rede D'Or São Paulo e Hospital Copa Star, Rio de Janeiro, RJ, Brasil.; 6 Universidade de São Paulo Faculdade de Medicina Hospital das Clínicas São Paulo SP Brasil Divisão de Cirurgia Torácica, Instituto do Câncer do Estado de São Paulo, Hospital das Clínicas, Faculdade de Medicina da Universidade de São Paulo, São Paulo, SP, Brasil.; 7 Instituto Oncoclínicas, Pesquisa e Educação Divisão de Oncologia Rio de Janeiro RJ Brasil Divisão de Oncologia, Instituto Oncoclínicas, Pesquisa e Educação, Rio de Janeiro, RJ, Brasil.; 8 Universidade de São Paulo Faculdade de Medicina Hospital das Clínicas São Paulo SP Brasil Divisão de Patologia, Hospital das Clínicas da Faculdade de Medicina da Universidade de São Paulo, São Paulo, SP, Brasil.; 9 Rede D'Or Rio de Janeiro e Hospital Copa Star Unidade de Cirurgia Torácica Rio de Janeiro RJ Brasil Unidade de Cirurgia Torácica, Rede D'Or Rio de Janeiro e Hospital Copa Star, Rio de Janeiro, RJ, Brasil.; 10 Pontifícia Universidade Católica do Rio de Janeiro Unidade de Cirurgia Torácica Rio de Janeiro RJ Brasil Unidade de Cirurgia Torácica, Pontifícia Universidade Católica do Rio de Janeiro, Rio de Janeiro, RJ, Brasil.

## Abstract

Cyclic Cushing's syndrome (CS) due to thymic carcinoid is a rare disorder. We report a case of cyclic CS due to ectopic adrenocorticotropic hormone (ACTH)-secreting atypical thymic carcinoid tumor and reviewed similar cases published in the literature. Our patient had hypercortisolemia lasting approximately one month, followed by normal cortisol secretion, with relapse one year later. Histopathology revealed an atypical ACTH-positive thymic carcinoid. Ectopic CS can be derived from atypical thymic carcinoids, which can be aggressive tumors with early relapse, suggesting that this type of tumor probably needs aggressive treatment.

## INTRODUCTION

Cushing's syndrome (CS) is a rare disorder characterized by inappropriately elevated secretion of cortisol ( 1 ). The syndrome has an estimated annual incidence of 0.2 to 5 per million persons ( 2 ). Most cases are caused by the overproduction of adrenocorticotrophic hormone (ACTH) by a pituitary corticotropinoma ( 2 ). Approximately 10% of CS cases result from the ectopic secretion of ACTH and are mainly derived from the foregut (larynx, thymus, lungs, stomach, duodenum and pancreas) ( 3 , 4 ). Approximately 100 cases of CS arising from thymic neuroendocrine tumor (NET) have been described to date ( 2 , 5 ). In rare cases, these thymic NET can be associated with cyclic CS ( 1 ).

We report a case of cyclic CS caused by a thymic NET and review similar cases published in the literature.

## CASE REPORT

A 32-year-old Caucasian man presented with sudden and unexplained weight gain (nine kilograms in a month), decreased libido, arterial hypertension and acne at 30 years of age, which resolved spontaneously. One year later, he developed the same rapid-onset signs and symptoms, along with severe anxiety and panic attacks that required clonazepam treatment. At this time, the patient was referred to our institution, and we noted in retrospect that he had been investigated for decreased libido at the age of 29 years, being diagnosed and treated for central hypogonadism [total testosterone: 171 ng/dL [reference value (RV): 220-819; FSH: 5.2 mUI/mL (RV: 1.5-12.4); LH: 3.5 mUI/mL (RV: 1.7-8.6); prolactin: 7.3 ng/mL (RV: 2.0-15.2)] with no further investigation. Physical examination revealed a typical cushingoid appearance, with facial plethora, moon face, central obesity, acne on his thighs, arterial hypertension and mild tachycardia. Cushing's syndrome was investigated, and biochemical assessment was compatible with ACTH-dependent CS: urinary free cortisol (UFC): 8077.5 mcg/24 hours (RV: 21-111); 11 p.m. salivary cortisol: 8.3 mcg/dL (RV: <0.2); ACTH: 178 and 264 pg/mL (RV: < 46); 8 a.m. basal cortisol: > 60 and 119.1 mcg/dL. The dehydroepiandrosterone sulfate level was 1947 mcg/dL (RV: 99-449), and the potassium level was normal at 4.6 mEq/L (RV: 3.5-5.3). He also showed central hypogonadism: total testosterone: 199 ng/dL (RV: 220-819); FSH: 2.1 mUI/mL (RV: 1.5-12.4); LH: 1.9 mUI/mL (RV: 1.7-8.6).

**Figure 1:**
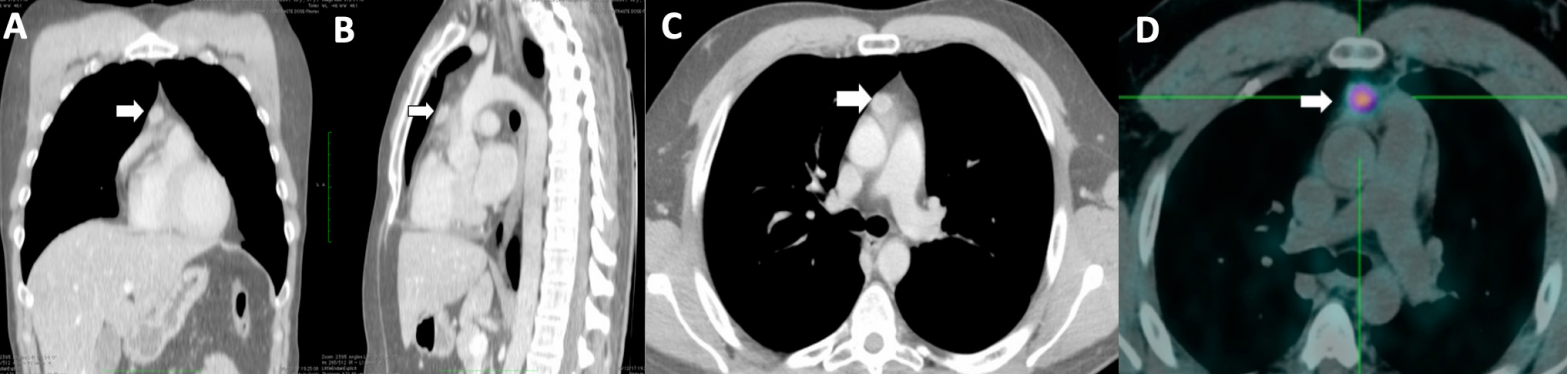
Chest computed tomography scan in the coronal ( **A** ), sagittal ( **B** ) and axial ( **C** ) views showing a 2.0×1.1×1.4 cm tumor in the anterior mediastinum (arrows) and ^68^Ga-DOTATOC-PET CT scan in the axial view (D) exhibiting focal radiotracer uptake in the anterior mediastinum (SUVmax = 6.7/15×11 mm).

No pituitary lesion was observed on magnetic resonance imaging (MRI). Bilateral inferior petrosal sinus sampling (BIPSS) demonstrated an ectopic origin for the hypercortisolemia (the ratio between central and peripheral ACTH was 1.2 at baseline and 2.3 after 10 mcg of endovenous desmopressin). BIPSS was performed when the patient was likely exciting the active phase and entering normocortisolemia, as suggested by 8 a.m. basal cortisol value of 14.6 mcg/dL and still increased ACTH levels of 54.9 pg/mL measured six days after the biochemical confirmation of ACTH-dependent CS as stated above. UFC was not measured on the day of BIPSS. Even done at this stage, BIPSS was compatible with an ectopic origin (the gradient between central and peripheral ACTH was <2 at baseline and <3 after desmopressin). Computed tomography (CT) of the chest revealed an anterior mediastinal mass suggesting a thymic lesion ( Figure 1 ). Octreotide receptor scintigraphy (octreoscan) demonstrated increased uptake of the tracer in the anterior mediastinum. The patient showed no evidence of carcinoid syndrome. He had normocalcemia, a normal chromogranin A level of 2.4 nmol/L (RV: < 3.0) and a negative family history of multiple endocrine neoplasia type 1 (MEN1).

Similar to the previous episode, the signs and symptoms of CS resolved spontaneously after 30 to 40 days, with significant weight loss of 10.5 kg in this period and the 8 a.m. cortisol levels decreasing to 10.3 mcg/dL, suggesting cyclic CS. He showed no clinical or biochemical evidence of adrenal insufficiency. The central hypogonadism at the age of 29 years was attributed to inhibition of the hypothalamus-pituitary-gonadal axis due to the hypercortisolemia that was likely already present at this age.

**Figure 2:**
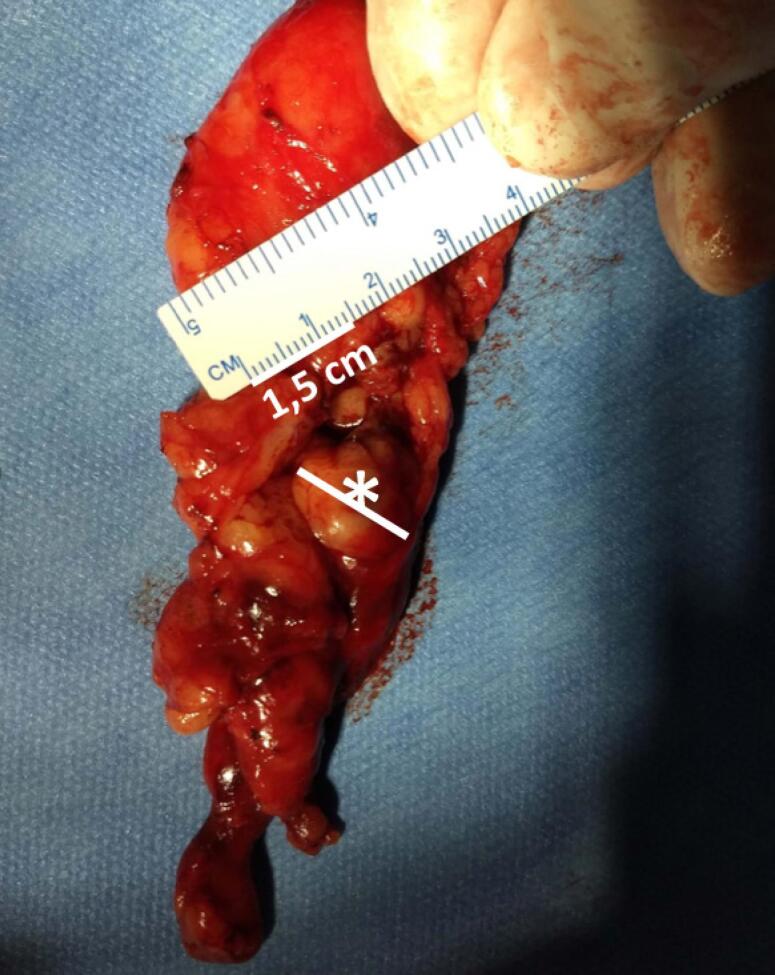
Surgical specimen with the tumor (*) measuring 1.5 cm with the thymic remnant showing several carcinoid tumorlets in the surrounding abundant fat.

**Figure 3:**
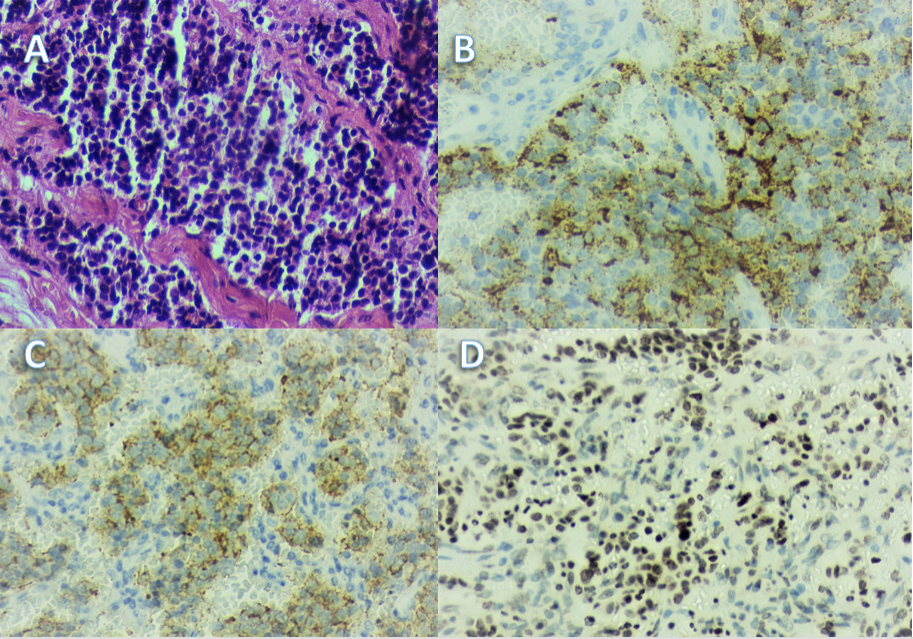
Histopathology of atypical thymic carcinoid. ( **A** ) Hematoxylin-eosin staining, ×200, showing a solid and trabecular growth pattern among a delicate vascularized fibroconjunctival stroma; ( **B** ) Immunohistochemistry, ×200, showing granular membranous ACTH staining; ( **C** ) Immunohistochemistry, ×200, showing strong membranous chromogranin A staining; ( **D** ) Immunohistochemistry, ×200, showing a high Ki67 proliferation index.

Robotic thymectomy was performed with a surgical description of complete tumor removal. The surgical specimen showed a tumor of 1.5 cm and several carcinoid tumorlets (<1 cm) in the surrounding abundant fat ( Figure 2 ). Microscopically, the tumor was comprised of uniform cells with nested, trabecular, and rosette-like growth patterns. The polygonal tumor cells had moderate eosinophilic granular cytoplasm, round to oval nuclei, “salt and pepper” chromatin and inconspicuous nucleoli ( Figure 3A ). Large zones of necrosis were present, and the mitotic count varied from 4 to 6 mitoses/mm^2^. Immunohistochemistry of the tumor samples was positive for CD56, chromogranin A, synaptophysin, and ACTH, and the Ki-67 index greater than 35% ( Figures 3B-D ).

On postoperative assessment, the patient required glucocorticoid coverage for a short period (1.5 months). The ACTH level was normalized to 43.6 pg/mL. After three months of surgery, he had normal free urinary cortisol and 11 p.m. salivary cortisol but nonsuppression in an overnight dexamethasone suppression test [serum cortisol: 2.6 mcg/dL (RV: <1.8)]. The chromogranin A levels increased to 3.3 nmol/L (RV: <3.0), and ACTH increased to 71.6 pg/mL four months after surgery. At this time, the ^68^Ga-DOTATOC-PET CT scans demonstrated focal uptake in the anterior mediastinum ( Figure 1 ), with mild radiotracer uptake in the same location as ^18^F FDG-PET CT. Although the patient had undergone complete surgical resection and remained asymptomatic, an increase in ACTH, chromogranin A and focal uptake in PET CT scans may indicate early relapse of an aggressive tumor. He remains on hypertensive medications, and has normal glycemic levels and a normal bone mineral density.

Considering the above findings, the patient was diagnosed with cyclic CS due to ACTH-secreting atypical thymic carcinoid based on the 2015 World Health Organization classification for neuroendocrine neoplasms.

## DISCUSSION

We report a case of a male patient who presented with rapid-onset symptoms suggestive of hypercortisolemia that occurred during cycles that lasted approximately one month and relapsed every year for two years before the diagnosis of ectopic CS due to atypical thymic carcinoid. Approximately 100 cases describing CS resulting from ACTH secretion from thymic tumors have been reported in the literature, with fewer than 10 cases of cyclic CS due to ectopic ACTH secretion from a thymic NET ( 1 , 5 - 11 ).

Thymic NET are rare tumors with an annual incidence of 0.07 to 0.18 per million persons, with higher rates in Caucasians and male individuals ( 12 ). However, thymic ACTH-producing NET exhibit no gender preference and are usually diagnosed in early adulthood (21-35 years of age) ( 13 ). Functionally active tumors are seen in one-third to half of cases ( 13 ). Thymic carcinoids are associated with MEN 1 in up to 25% of cases ( 13 ).

In ectopic ACTH secretion, the ACTH plasma levels are usually very high, as observed in our case ( 1 ). Because most NET express somatostatin receptors, octreotide receptor scintigraphy (octreoscan) or PET CT with ^68^Gallium-labeled somatostatin analogs can be used to evaluate these patients, including in cases of ectopic ACTH-secreting tumors ( 14 - 16 ).

NET produce biogenic amines such as serotonin which may lead to carcinoid syndrome. NET originate from one of three portions of the primitive gut: foregut, midgut and hindgut. Thymic NET are foregut tumors, which have lower serotonin metabolism than midgut tumors, reflecting a low frequency of carcinoid syndrome in these patients, as observed in our case ( 1 ).

Patients with CS (from different etiologies) have episodic cortisol secretion ( 17 ). However, in a small subset of CS patients, highly variable levels of glucocorticoid secretion can occur. Episodes of hypercortisolemia interspaced with periods of normocortisolemia (or adrenal insufficiency) are known as cyclic CS ( 1 ). Adrenal insufficiency can also be observed, but was not seen in our case, probably because of the short cycle length. In cyclic CS, rhythmic fluctuations in ACTH secretion occur and result in more or less predictable cyclic variation in adrenal steroid production ( 18 ). The cycle length usually lasts one month and the intercyclic phase is prolonged in ectopic secretion of ACTH, as seen in our case ( 1 ). Meinardi and cols. ( 1 ) reviewed 65 cases of cyclic CS cases. Most of the cases were due to Cushing's disease (CD), followed by the ectopic secretion of ACTH in 26% of cases. Considering that approximately 10% of CS cases are due to ectopic ACTH secretion, cyclic CS seems to occur more frequently in patients with ectopic ACTH secretion ( 1 ).

Based on literature reports of thymic NET causing cyclic CS published to date, the epidemiology is similar to thymic NET causing CS. The diagnosis usually occurs in early adulthood (20-43 years old), with no gender predilection ( 1 , 5 - 9 , 11 , 13 ). Nevertheless, one case was described in a 7-year-old child ( 10 ). CS cycles may last days or months or are seasonal, as described by Trott and cols. ( 5 ). Additionally, symptoms of hypercortisolemia may not be typical because of the cyclic nature of the ACTH production. Furthermore, hypokalemia and osteoporosis, which are more frequent in ectopic CS, are not always present in cyclic CS and was not observed in our case ( 3 ). Nonetheless, cortisol-induced comorbidities should be monitored regularly. Cyclic CS also seems to cause more psychiatric disturbances than noncyclic CS, as observed in our case.

Cyclic CS is difficult to diagnose, requiring clinical suspicion and repeated testing. Dynamic testing is often inconclusive due to unsustained hypercortisolemia. The mechanism underlying cyclic CS remains unclear. A proposed explanation would be episodes of spontaneous tumor hemorrhage or cyclic growth and apoptosis of ACTH-secreting tumor cells ( 19 , 20 ).

The 2015 WHO classification of thymic neuroendocrine tumors is generic but accurate, and the lesions are classified into three grades according to the mitotic count and presence of necrosis ( 21 ). Low-grade (typical carcinoid) lesions have 2 or fewer mitotic counts and no necrosis, intermediate-grade (atypical carcinoid) lesions have 2-10 mitotic counts and foci of necrosis, and high-grade (large cell neuroendocrine carcinoma and small cell carcinoma) lesions have more than 10 mitotic counts and the presence of necrosis. The distinction between an atypical neuroendocrine tumor (AC) and a large cell neuroendocrine carcinoma (LCNEC) is difficult based solely on the 2015 WHO pathological classification. Other pathological classifications and clinicopathological correlations are required. Applying the Ki-67-based ENETS classification, which shows that the Ki-67 index of AC varies on average from 1% to 18.8% and that of LCNEC varies from 16% to 90%, our case would be considered LCNEC ( 22 , 23 ). However, based on early relapse, mitotic count and high Ki-67 index, the tumor was classified as overlapping between AC and LCNEC and considered an aggressive atypical neuroendocrine tumor.

Surgical treatment, with complete tumor removal, is the treatment of choice. Despite aggressive treatment, thymic NET have a poor prognosis. The ten-year survival rate is 38%, with worse outcomes in patients with CS ( 24 - 26 ). The role of chemotherapy has not been well established because the low number of studied patients limits its assessment ( 1 ).

Despite the resolution of hypercortisolemia with the resection of thymic tumors in our case, the patient had an ACTH level in the upper limit of the normal range and a nonsuppression response to the overnight dexamethasone suppression test, which could represent an unfavorable prognosis.

In conclusion, cyclic CS represents a clinical challenge requiring clinical suspicion. Ectopic CS can be derived from atypical ACTH-producing thymic carcinoids, which can relapse early, even after complete surgical removal. This outcome shows that this type of aggressive disease likely requires aggressive treatment.
